# Case Report of a Patient Presenting with Nonketotic Hyperglycemia Hemichorea

**DOI:** 10.21980/J8.52115

**Published:** 2025-10-31

**Authors:** Jay Patel, Kayla Pena, Joshua Bucher, Amanda Esposito

**Affiliations:** * Rutgers Robert Wood Johnson Medical School, Department of Emergency Medicine, New Brunswick, NJ

## Abstract

Nonketotic hyperglycemia hemichorea is a rare neurological manifestation associated with uncontrolled diabetes mellitus. This case report presents the clinical features, diagnostic workup, and management of a 74-year-old female with hemichorea secondary to nonketotic hyperglycemia. Patient presented to the emergency department with acute onset of right leg movement that was non-purposeful and random without any associated neurological symptoms such as paresthesias weakness, pain, or systemic symptoms. Patient’s medical history included untreated diabetes mellitus with glucose of 198 measured in the ED. Computed tomography of the and neck were unremarkable for any signs of ischemia, occlusion, hemorrhage or masses. Brain MRI without contrast showed T1 shortening within the left basal ganglia involving both the caudate nucleus and the lentiform nucleus, a radiologic finding common in nonketotic hyperglycemia hemichorea. Patient was admitted to the hospital for diabetes management. Patient’s symptoms did not completely resolve prior to discharge from the hospital but was closely monitored by her primary care doctor and neurologist; she had significant improvements in her symptoms over the subsequent year with improvement in her activities of daily living (ADLs).

**Topics:**

Nonketotic hyperglycemia hemichorea, diabetes, hemichorea.

**Figure:**
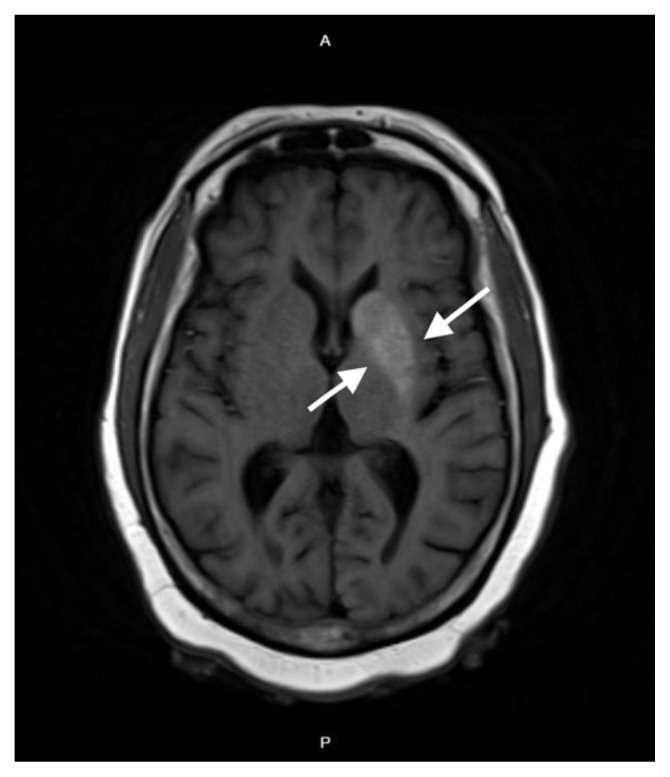


## Brief introduction

Nonketotic hyperglycemia hemichorea is characterized by choreiform movements affecting one side of the body, primarily associated with hyperglycemia in the absence of ketoacidosis. It is estimated to have a prevalence of one in 100,000 in diabetic patients.^1^ It is characterized by involuntary movements known as chorea or ballism with distinct changes in the basal ganglia, observable through CT or MRI scans.^2^ This neurological manifestation is rare but significant, necessitating early recognition and intervention for optimal outcomes. The link between hyperglycemia and chorea was initially proposed by Lin and Lin in 1968, and subsequent studies have elucidated the intricate pathophysiological mechanisms.^3^ The prevailing hypothesis suggests that hyperglycemia leads to alterations in the basal ganglia, a region crucial for motor control, resulting in the characteristic involuntary movements seen in non-ketotic hyperglycemia hemi-chorea.^4^

## Presenting concerns and clinical findings

Mrs. Doe is a 74-year-old female who presented with several hours of involuntary, jerky movements affecting her right leg that were not suppressible and continued even with voluntary movement. She had no other associated symptoms including leg pain, weakness, paresthesias, slurred speech, facial droop, headache, vomiting, or dizziness. The patient’s medical history was pertinent only for a history of diabetes, but she has not been on any medication for the past few years. On exam, she was alert and oriented to name, place, and time, with no aphasia, cranial nerves II–XII intact, sensation intact, motor strength 5/5 in all extremities. She had choreiform movements in right lower extremity but no drift was present. There were no other pertinent positive findings on exam. A stroke alert was initiated at our institution based upon the findings.

## Significant findings

Laboratory tests indicated elevated blood glucose levels (198 mg/dL) with no urinary ketones, anion gap of 12, thyroid stimulating hormone (TSH) of 12 UIU/ml, and an increased glycated hemoglobin (HbA1c) of 14.9%. After initial stroke evaluation with neurology, imaging studies, including computed tomography (CT)/CT angiography (CTA) of the brain and neck, were unremarkable, ruling out structural lesions or acute stroke. Neurology recommended an MRI which showed T1 shortening within the left basal ganglia involving both the caudate nucleus and the lentiform nucleus. T1 shortening indicates changes in the tissue composition or structure that alters how the tissue responds to the MRI pulse, giving the tissue a brighter appearance on MRI (see white arrow).

## Patient course

The patient was admitted for glycemic control with insulin therapy. Neurology recommended tetrabenazine to help resolve hemichorea symptoms. During her hospitalization, she continued to have uncontrollable right lower leg movements. No additional imaging was performed while hospitalized. After glucose was controlled with an insulin sliding scale, she was transitioned to a basal insulin and Metformin. The patient was discharged with close outpatient follow-up for continued diabetes management and neurological monitoring. At a tele-visit one week after discharge she was still found to have right lower extremity movements but no other complaints such as weakness, numbness, disorientation or ambulatory dysfunction. She continued taking metformin 500mg twice a day and 10 units of lantus in the evening.

Five months after her initial hospitalization, she had an outpatient follow up with neurology where she reported a 50% reduction in symptoms; however, she still struggled with chorea symptoms affecting activities of daily living. Patient was prescribed tetrabenazine which she took for one month but then discontinued it due to personal financial concerns. There was an overall improvement in symptoms after better glycemic control and physical therapy.

## Discussion

Nonketotic hyperglycemia hemichorea (NKHH) is characterized by choreiform movements affecting one side of the body and is primarily associated with hyperglycemia in the absence of ketoacidosis. This neurological manifestation is rare but significant, necessitating early recognition and intervention for optimal outcomes. The differential diagnosis for a patient presenting with choreiform movements is broad and includes ischemic or hemorrhagic stroke, seizures with postictal movement disorders, primary movement disorders such as Huntington’s disease or Wilson’s disease. Other considerations include drug-induced dyskinesias (eg, from antipsychotics or antiemetics), structural brain lesions (eg, tumors or abscesses), and infections such as Sydenham chorea or autoimmune encephalitis.^5^

In nonketotic hyperglycemia hemichorea, the pathogenesis is multifactorial and not yet fully understood, though several mechanisms have been proposed. The most widely accepted theory involves metabolic dysfunction within the basal ganglia due to hyperglycemia-induced impairment of GABA synthesis, leading to disrupted inhibitory signaling and resultant involuntary movements.^4^ Additionally, hyperviscosity and dehydration may contribute to reduced perfusion and microvascular compromise in the striatum, potentially causing transient ischemia or microhemorrhages, which correlate with the characteristic T1 hyperintensities seen on MRI.^6^ Disruption of the blood-brain barrier and subsequent intracellular acidosis may further exacerbate neuronal dysfunction.^7^ While these mechanisms help explain the clinical and radiographic findings seen in NKHH, further research is needed to better define the pathophysiological processes involved.

Neuroimaging plays a critical role in diagnosing NKHH, with MRI being the most sensitive modality. Characteristic findings on T1-weighted MRI include hyperintense lesions in the contralateral basal ganglia—typically the putamen and caudate nucleus—seen in the majority of reported cases.^4^ While exact sensitivity and specificity values are not well quantified due to the rarity of the condition, several case series and reviews report MRI abnormalities in nearly all confirmed cases of NKHH, making it the preferred imaging modality for diagnosis.^4^ Non-contrast CT may reveal hyperdensities in the same regions, though it is less sensitive and can be misinterpreted as hemorrhage or calcification.^8^ In some instances, CT findings may be normal, further emphasizing the value of MRI.^9^ The reliance on MRI underscores the importance of early advanced imaging in patients presenting with unilateral choreiform movements and hyperglycemia, especially when initial CT findings are equivocal.

In this case, the patient exhibited classic hemichorea symptoms associated with nonketotic hyperglycemia, but the patient’s symptoms persisted despite glycemic control. This highlights the variability in symptom resolution, which can range from a few hours to weeks, as seen in other reported cases.^10^,^11^ Effective blood sugar management generally leads to a favorable prognosis; however, symptom resolution is not always immediate post-treatment.^12^ In addition to glucose control, patients are often prescribed antipsychotics, like haloperidol or tetrabenazine, to regulate dopamine levels and basal ganglia activity, further emphasizing the complexity and variability in managing this condition.^1^

The rarity of this presentation often leads to either a missed diagnosis or misdiagnosis. For emergency medicine physicians, early diagnosis of NKHH is crucial to ensure the initiation of early strict glucose control. It can be misdiagnosed with other conditions such as stroke, Parkinson’s disease, seizure, psychogenic movement disorder, drug-induced movement disorders, and peripheral neuropathy. Major consequences of misdiagnosis are delayed treatment and inappropriate interventions. One such inappropriate intervention may be giving tenecteplase (TNK) for a presumed stroke, which could cost $2,000–5,000 leading to increased healthcare costs for the patient as well as bleeding complications from the intervention. Moreover, early diagnosis allows for timely correction of hyperglycemia, which remains the cornerstone of management and is associated with rapid clinical improvement in most cases. Delayed diagnosis, by contrast, can result in prolonged symptoms, avoidable hospital admissions, and increased morbidity. Therefore, familiarity with NKHH is essential in the emergency setting, especially given its status as a rare but reversible stroke mimic.

## Supplementary Information





## References

[1] Chua CB, Sun CK, Hsu CW (2020). “Diabetic striatopathy”: clinical presentations, controversy, pathogenesis, treatments, and outcomes. Sci Rep.

[2] Lau SC, Tan SM (2023). Nonketotic Hyperglycemic Hemichorea. Am J Med.

[3] Lin JJ, Lin GY (2007). Non-ketotic hyperglycemia-related hemichorea/hemiballism. Parkinsonism Relat Disord.

[4] Oh SH, Lee KY, Im JH, Lee MS (2002). Chorea associated with non-ketotic hyperglycemia and hyperintensity basal ganglia lesion on T1-weighted brain MRI study: a meta-analysis of 53 cases including four present cases. J Neurol Sci.

[5] Roy U, Das SK, Mukherjee A (2016). Irreversible hemichorea-hemiballism in a case of nonketotic hyperglycemia presenting as the initial manifestation of diabetes mellitus. Tremor Other Hyperkinet Mov (N Y).

[6] Lee D, Lee YB, Lee YS (2002). Hemichorea associated with nonketotic hyperglycemia: clinical characteristics, radiological findings, and treatment outcomes. J Neurol.

[7] Chang KH, Tsou JC, Chen ST, Ro LS, Lyu RK, Chan WP (1996). Temporal features of striatal abnormalities in nonketotic hyperglycemia: diffusion-weighted MRI in chorea. AJNR Am J Neuroradiol.

[8] Shan DE, Ho DM, Chang C, Pan HC, Teng MM (1998). Hemichorea-hemiballism: an explanation for MR signal changes. AJNR Am J Neuroradiol.

[9] Hwang KJ, Park MH, Kim M, Park KP (2014). Hemichorea-hemiballismus in non-ketotic hyperglycemia: MR imaging findings and a review of literature. J Neurol Sci.

[10] Priola AM, Gned D, Veltri A (2014). Case 204: nonketotic hyperglycemia-induced hemiballism-hemichorea. Radiology.

[11] Ahlskog JE, Nishino H, Evidente VG (2001). Persistent chorea triggered by hyperglycemic crisis in diabetics. Mov Disord.

[12] Awasthi D, Tiwari AK, Upadhyaya A, Singh B, Tomar GS (2012). Ketotic hyperglycemia with movement disorder. J Emerg Trauma Shock.

