# Supplementary figures and images for: Case Report of a Patient Presenting with Nonketotic Hyperglycemia Hemichorea
Source: J Educ Teach Emerg Med. 2025 Oct 31;10(4):V1–4. doi: 10.21980/J8.52115 (PMC12594471; doi:10.21980/J8.52115)

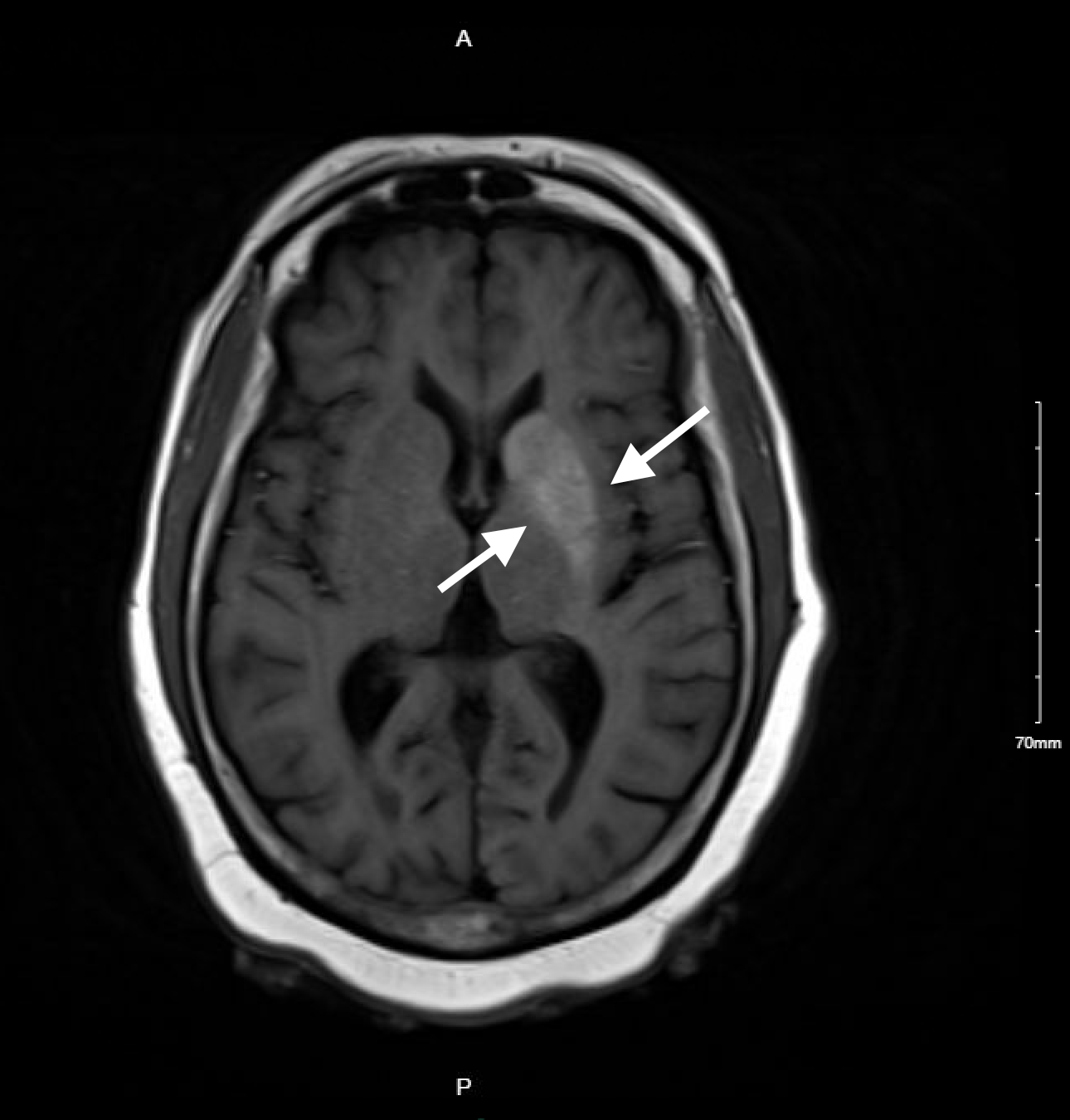

Supplement: Supplementary file 1 [file 10-4-V1-Supp1.png]

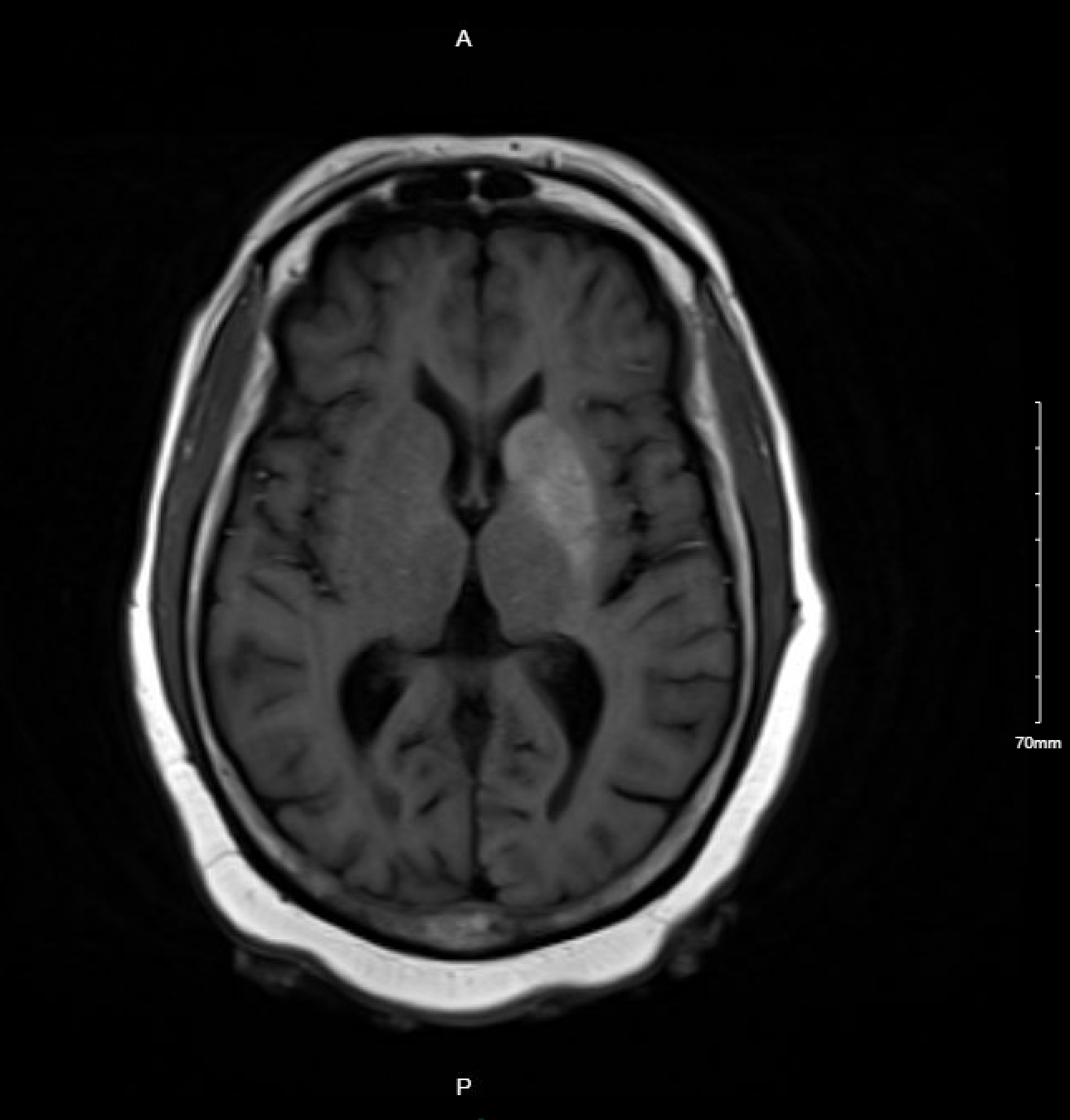

Supplement: Supplementary file 2 [file 10-4-V1-Supp2.png]
